# Internal Medicine Training in Ethiopia: Honoring the Legacy, Embracing the Future

**DOI:** 10.4314/ejhs.v35i4.1

**Published:** 2025-07

**Authors:** Abel Mureja, Abate Bane, Wondwossen Amogne, Yeweyenhareg Feleke, Ahmed Reja

**Affiliations:** 1 St. Paul Hospital Millenium Medical College, Department of Internal Medicine, Addis Ababa, Ethiopia; 2 Addis Ababa University, Department of Internal Medicine, Addis Ababa, Ethiopia

Internal medicine was the first science based clinical specialty and also established the three fundamental pillars of modern medicine: clinical practice, research and medical education. Internal Medicine is a core medical specialty defined by a holistic, patient centered approach (1). The origins of Internal medicine as distinct specialty are rooted in the late 19th Century. The term Internal medicine itself was adopted from German term “innere Medizine. In the United States, William Osler, a Canadian born physician is considered a seminal figure in the development of Internal medicine.

The history of internal medicine training in Ethiopia involves significant development and ongoing efforts to establish a strong and sustainable program. From its modest beginnings in the 1960s to today's expanding academic landscape, the development of internal medicine education has paralleled the country's broader pursuit of health system self-reliance and professional excellence.

It began in 1964 with the founding of the Department of Internal Medicine at Addis Ababa University, led at the time by committed expatriate faculty. The official launch of residency training in 1979 represented a significant milestone, establishing the groundwork for developing a locally trained community of specialists as the department welcomed its first cohort of 11 graduate students (2). Professor Edemariam Tsega served as the inaugural Head of the Department from March 1974 to August 1990 (3). Those early years were demanding, shaped by limited resources but rich in clinical experience. Training was primarily conducted through bedside mentorship, with few textbooks accessible and journal articles typically delivered by mail. Yet within this environment, the defining values of Ethiopian internal medicine emerged: strong clinical reasoning, ethical integrity, and a deep sense of professional purpose.

Internal medicine training is now offered at numerous sites across Ethiopia, with over 20 medical schools enrolling hundreds of residents (4). Teaching remains heavily traditional, focused on didactic lectures, ward rounds, and journal discussions. However, the system faces challenges such as limited diagnostics and therapies, faculty shortages, and underfunding that hinder mentorship and supervision(5). Communication skills, professionalism, and structured, competency-based assessment tools are inconsistently integrated.

Reform efforts continue to advance within the field. Globally, internal medicine education is shifting to incorporate technology-enhanced, competency-based, and team-oriented approaches(6). Embracing digital platforms, simulation labs, clinical decision tools, and digital health technologies can expand access, improve training quality, and elevate healthcare delivery. Incorporating artificial intelligence and digital health tools into internal medicine training and service is also essential to keep pace with the ever-evolving global landscape of medical education and care. Strategic international collaborations and curriculum accreditation can raise training standards and enhance global competitiveness. Equally important is embedding research, leadership, communication skills, and career development into residency programs—nurturing future internists who will lead change, not merely deliver care.

Central to this reform is a stronger commitment to supporting both residents and faculty. Housing, fair pay, and protected time for teaching are not luxuries; they are necessities (5). Professional societies must take the lead in shaping curricula, overseeing the quality of training and health care services, upholding ethical standards, and advocating for improved working and living conditions for health professional as well as enhanced access to essential diagnostics and therapeutics.

The pioneers who laid the foundation for internal medicine training in Ethiopia built more than programs; they built a culture rooted in excellence despite adversity. As the system grows, it must not only carry that legacy forward but adapt it to the future. Now is the time to move from resilience to innovation. Emphasizing the education of physicians to actively contribute to the evolution of the medical system is essential, rather than limiting their training to adapting within established frameworks.

Internal medicine training in Ethiopia is confronted with significant challenges, including an increasing burden of chronic diseases and heightened patient demands. Nevertheless, through coordinated reform efforts and a revitalized strategic vision, it can remain a foundational element of equitable and effective healthcare delivery. Prompt action is essential, as the steps taken at this juncture will shape future outcomes.
